# Rapid clearance of facial verruca plana with local hyperthermia treatment combined with topical interferon alpha-2b in 3 weeks: a case report

**DOI:** 10.1186/s13256-026-05858-7

**Published:** 2026-02-25

**Authors:** Fang Liu, Rui-Qun Qi, Xuping Niu, Wenyu Wan

**Affiliations:** 1 Institute of Dermatology, Taiyuan Central Hospital of Shanxi Medical University, No.5 Dong San Dao Xiang, Jiefang Road, Taiyuan, 030009 Shanxi Province China; 2 Department of Dermatology, The First Hospital of China Medical University, 155N Nanjing Road, Heping District, Shenyang, 110001 China; 3 Key Laboratory of Immunodermatology, Ministry of Education, National Joint Engineering Research Center for Theranostics of Immunological Skin Diseases, and National Health Commission, Shenyang, China

**Keywords:** Facial warts, Verruca plana, Local hyperthermia, Interferon α-2b, HPV, Scar‑free therapy, Case report

## Abstract

**Background:**

Facial verruca plana can be therapeutically challenging and cosmetically distressing when extensive. Conventional ablative treatments (for example, cryotherapy) often carry risks of scarring and discomfort. We report a novel combination of local hyperthermia and topical interferon alpha-2b gel that achieved rapid, scar‑free clearance of widespread facial warts within 3 weeks.

**Case presentation:**

A 49‑year‑old Han Chinese male patient presented with over 60 verruca plana distributed across the cheeks, forehead, and nose. He had no history of immunodeficiency or systemic disease. Previous topical therapies yielded minimal improvement. The patient was treated with a pulsed regimen of local infrared hyperthermia (44 ± 2 °C) targeting representative lesions, combined with twice-daily topical application of interferon alpha-2b gel. By the sixth hyperthermia session (approximately 2 weeks after initiation), significant lesion regression was observed. Near‑complete clearance occurred by the tenth session (3 weeks), with no scarring or adverse effects. At the 16‑week follow‑up, there was no recurrence.

**Conclusion:**

The combination of targeted local hyperthermia and topical interferon alpha-2b gel appears to be a safe, effective, and cosmetically favorable treatment for extensive facial verruca plana. This regimen harnesses both immunomodulatory and antiviral mechanisms to accelerate viral lesion clearance while preserving skin integrity. Further studies in larger cohorts are warranted to validate this approach.

## Background

Generalized viral warts are common in immunocompromised patients and are extremely challenging in clinical practice owing to impaired immune function [1]. It has been reported that local infrared hyperthermia treatment at 44 ± 2 °C can noninvasively treat skin diseases caused by human papillomavirus (HPV) infection, such as verruca plana, plantar warts, and condyloma acuminate [2, 3]. The mechanism of this therapy might entail establishing a particular immune response against skin tissues infected with HPV, which can assist in eradicating HPV in irradiated and non-irradiated or distant areas [4].

Facial warts, in particular, pose a unique challenge owing to their visibility and the cosmetic concerns they raise, which can significantly affect a patient’s quality of life. Conventional treatments such as cryotherapy or electrosurgery often risk scarring or intense postprocedural discomfort—outcomes especially problematic when treating facial lesions, where cosmetic appearance is paramount. Consequently, there is a pressing need for therapeutic strategies that minimize tissue damage and optimize cosmetic results, ensuring not only the clearance of the warts but also the preservation of skin integrity. This case report presents an example of facial verruca plana treatment in which a minimally invasive approach was employed to address HPV infection while prioritizing aesthetic considerations and patient satisfaction.

## Case presentation

**Fig. 1:**
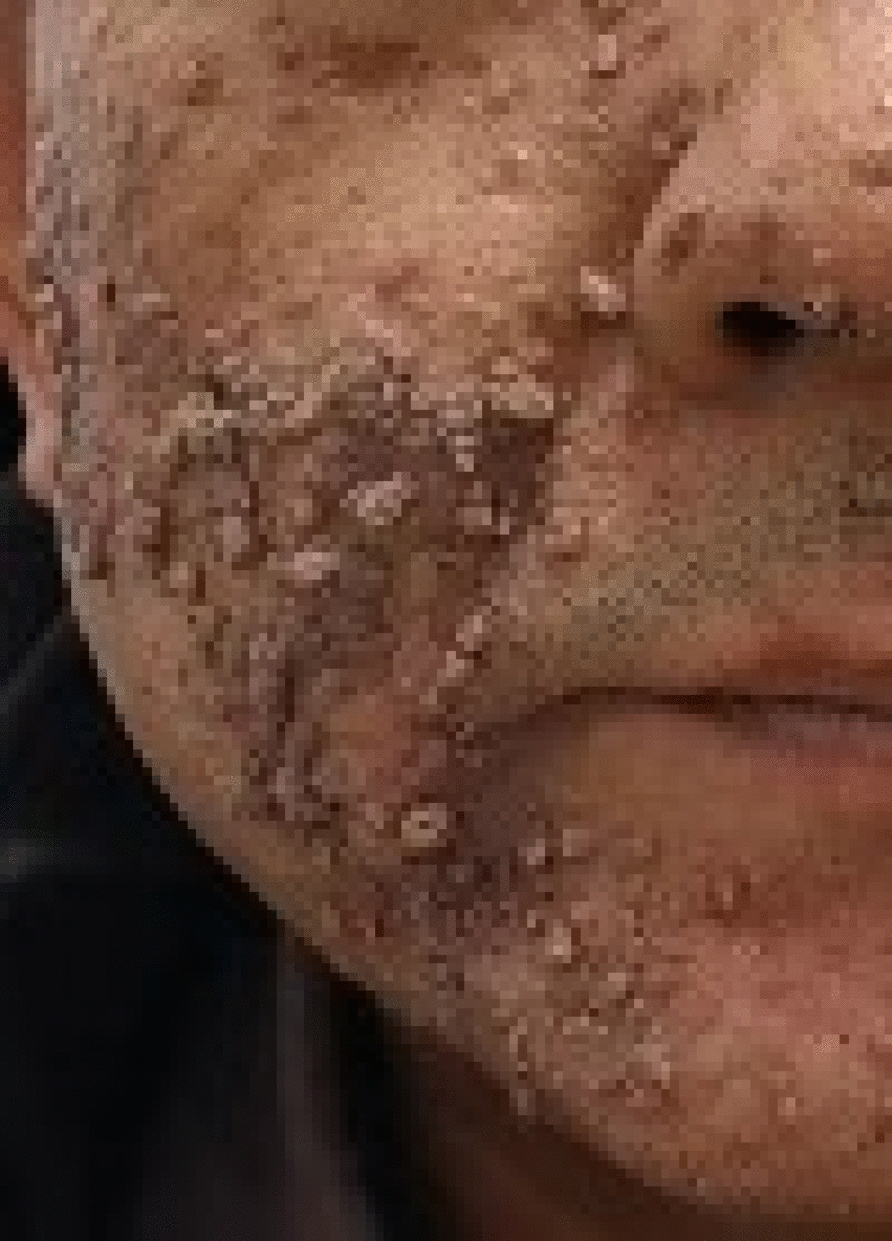
At the first visit, the patient presented with widespread facial common warts

**Fig. 2:**
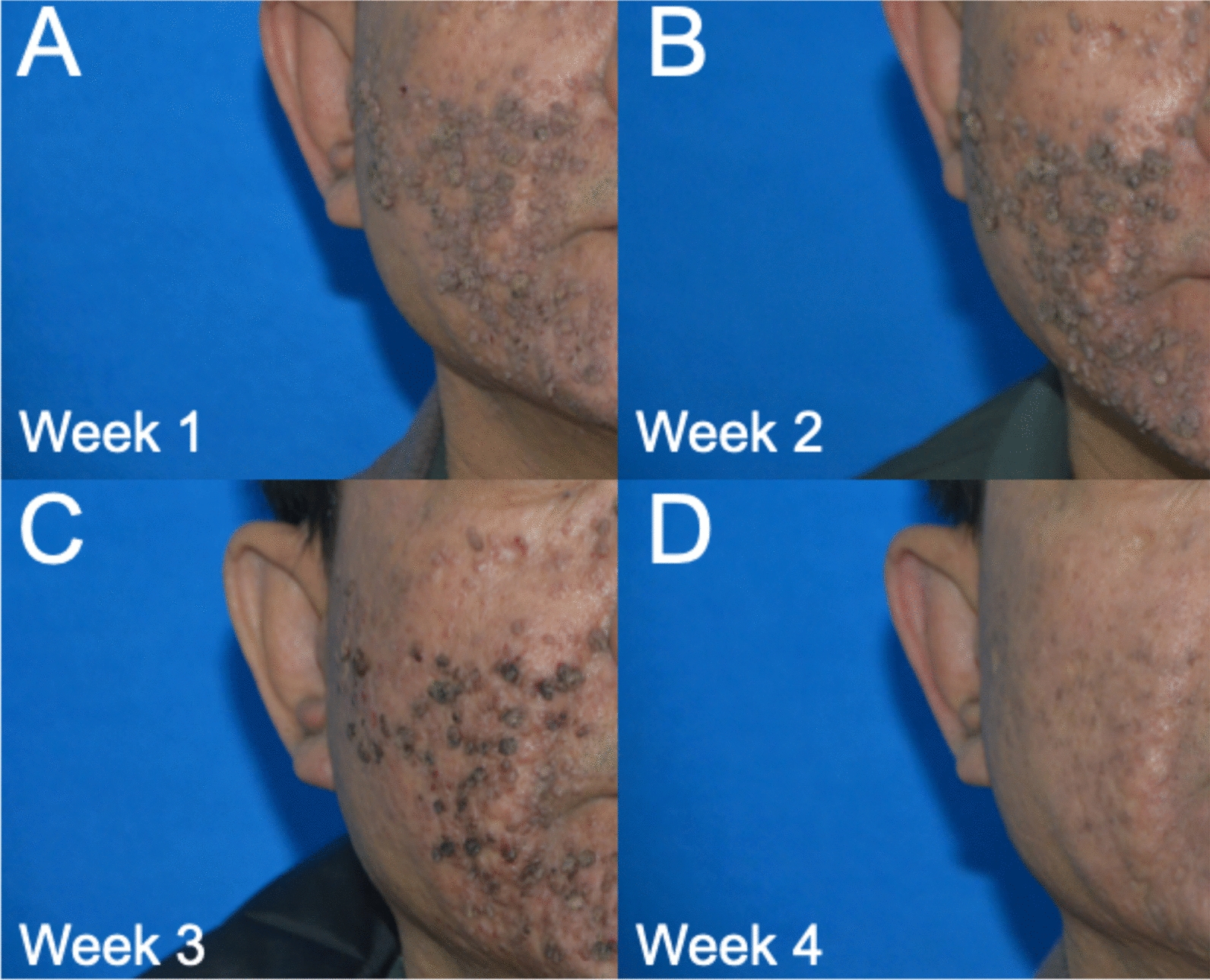
Clinical progression of facial verruca plana following standardized local hyperthermia treatment. The patient received a phased regimen consisting of: **A** three consecutive daily sessions during week 1; **B** two consecutive daily sessions during week 2; followed by **C**, a once-weekly 30-minute session during week 3, and **D** another weekly session in week 4. Visible improvement was observed by week 3, with near-complete clearance by week 4

**Fig. 3:**
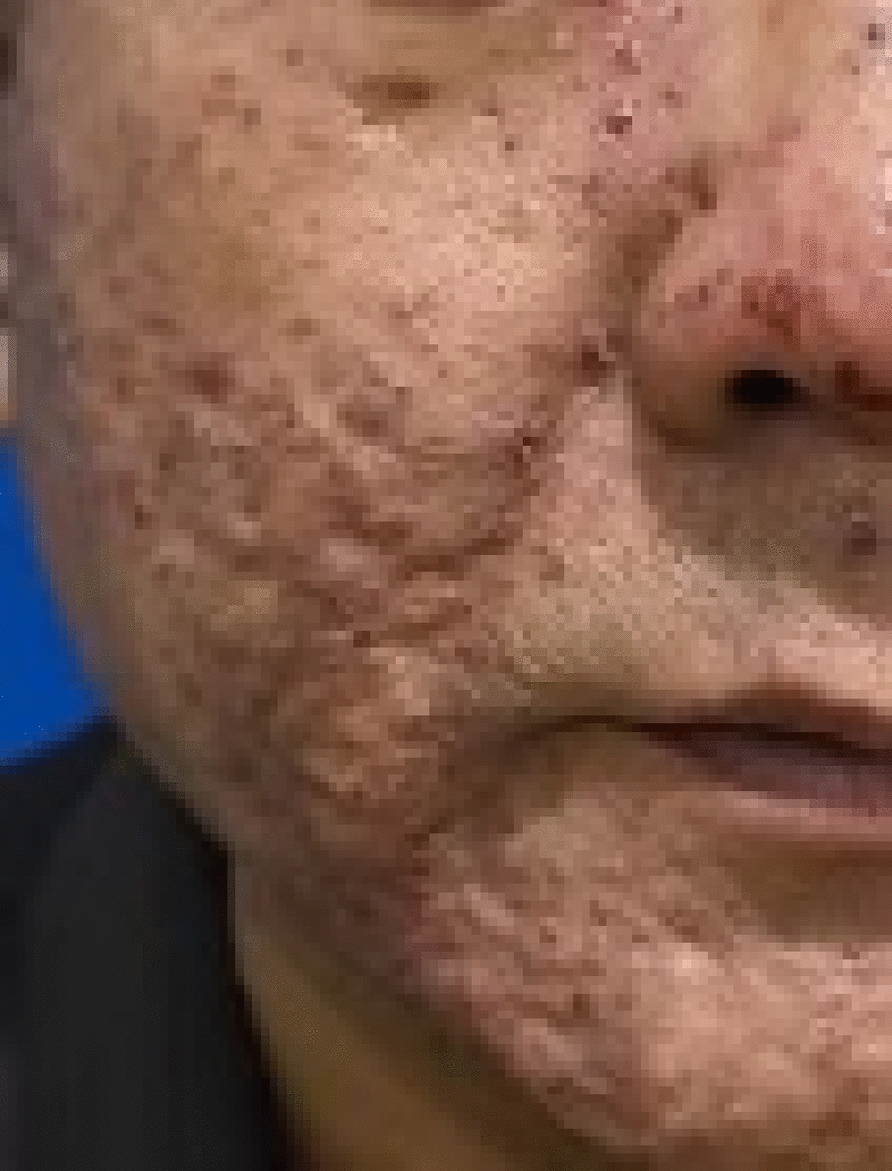
Following a 6-week observation period, all facial warts resolved completely without evidence of residual scarring or recurrence

We report a case of extensive facial verruca plana demonstrating rapid clearance of warts within 3 weeks, which was treated with local hyperthermia and topical interferon alpha-2b gel. A 49-year-old Han Chinese male patient presented with progressively increasing common warts across his face (Fig. 1). About 2 years ago, the patient developed a small number of verruca plana on the face. Without much success, he used topical *Brucea javanica*, a popular traditional Chinese medication that is effective for warts. The patient denied a history of immunodeficiency disease and was previously in good health. Given the widespread distribution of lesions on the patient’s face and the need to balance cosmetic outcomes with treatment tolerability, a treatment plan of local hyperthermia combined with topical interferon alpha-2b gel was initiated. The topical interferon gel for the face is applied twice daily. A standard hyperthermia treatment targeting HPV-associated cutaneous lesions was initiated. The treatment regimen consisted of three consecutive daily sessions (30-minute duration each), followed by a 6-day interval, then two consecutive daily sessions, and subsequently once-weekly sessions [5]. Clinical observation revealed gradual rash improvement, with initial subsidence noted following the sixth session (Fig. 2A, B). The patient’s warts started to recede in a large area at the sixth treatment (3 weeks after the first local hyperthermia treatment) (Fig. 2C). Near-complete resolution was achieved by the tenth treatment session (3 weeks after the first treatment) (Fig. 2D). The patient remained compliant with the prescribed regimen throughout the treatment course and experienced no adverse effects. Following a 6-week observation period, all facial verruca plana resolved completely without evidence of residual scarring (Fig. 3). There was no sign of recurrence after the 16-week follow-up.

## Discussion and conclusion

Using hyperthermia treatment alone to treat warts has a long course of treatment. In a randomized controlled trial that evaluated the clearance efficacy by applying local hyperthermia to a target lesion in patients with multiple warts, 54% achieved clearance of nontargeted lesions after a 4-month treatment [3]. The chance of immunological response to the infected keratinocytes may be increased by direct stimulation in the locality of the wart [1]. In a network meta-analysis, combining systemic retinoids with immunotherapy to treat warts can yield higher rates of complete clearance with lower recurrence [6]. To solve the problem of the slow effectiveness of hyperthermia alone, we adopted a treatment plan of hyperthermia combined with topical interferon gel.

The rapid clearance of facial lesions may result from the synergistic effects of local hyperthermia and topical interferon alpha-2b gel. Hyperthermia likely induces heat shock proteins that activate T cell responses and enhances skin permeability to improve drug delivery [7, 8]. Interferon may upregulate major histocompatability complex (MHC) class I expression and activate T cell-induced target cell lysis, promoting immune-mediated clearance [9]. In combination, hyperthermia helps “unmask” the virus, while interferon provides targeted antiviral and immunostimulatory signals.

Facial warts, while benign, often represent a significant cosmetic concern and can have a serious psychological impact—particularly when lesions are widespread [10], as in this case, involving over 60 warts across the cheeks, forehead, and nose. According to UK guidelines, cryotherapy combined with salicylic acid is the most recommended first-line treatment [1]. However, conventional therapies carry notable limitations: Cryotherapy often requires several months of repeated sessions and may lead to hypopigmentation or scarring; topical imiquimod typically takes 3–4 months and is frequently associated with irritation and erythema; pulsed dye laser treatment, while effective in some cases, is costly and can result in bruising. In contrast, our patient experienced near-complete clearance within just 3 weeks of initiating treatment with local hyperthermia combined with topical interferon alpha-2b gel—without pain, scarring, or adverse events. This case highlights the potential of this combination regimen as a rapid, well-tolerated, and cosmetically favorable alternative for the treatment of extensive facial verruca plana.

This case holds clinical significance for several reasons. First, it represents the first documentation of scar-free therapeutic resolution in extensive facial verruca plana using combination therapy. Second, it provides valuable clinical evidence supporting a scar-sparing alternative to conventional ablative modalities. Third, our findings suggest potential investigative pathways that can improve the insights of anti-HPV infection: (1) elucidating the immunomodulatory mechanisms underlying localized hyperthermia-induced HPV clearance and (2) determining whether adjuvant interferon alpha-2b gel application accelerates viral eradication through synergistic antiviral potentiation.

This case highlights the efficacy and safety of combining targeted local hyperthermia with topical interferon alpha‑2b for treating extensive facial verruca plana. The regimen achieved rapid, scar‑free lesion clearance within 3 weeks and demonstrated durable remission at 16 weeks without any adverse effects. By harnessing both thermal immunomodulation and antiviral activity, this approach offers a promising, cosmetically favorable alternative to conventional therapies. We hope this strategy will help others manage similar extensive lesions and demonstrate it in a larger cohort.

## Data Availability

Not applicable.

## Abbreviation

HPV: Human papillomavirus
